# Approaches, barriers, and facilitators to abortion-related work in U.S. health departments: perspectives of maternal and child heath and family planning professionals

**DOI:** 10.1186/s12889-020-8389-2

**Published:** 2020-03-06

**Authors:** Nancy F. Berglas, Erin Wingo, Katie Woodruff, Sarah C. M. Roberts

**Affiliations:** Department of Obstetrics, Gynecology and Reproductive Sciences, Advancing New Standards in Reproductive Health, University of California, San Francisco, 1330 Broadway, Suite 1100, Oakland, CA 94612 USA

**Keywords:** Abortion, Public health, Government agencies, Health policy

## Abstract

**Background:**

Public health agencies in the United States have engaged in abortion-related activities for nearly 50 years. Prior research indicates that, while most state health departments engage in some abortion-related work, their efforts reflect what is required by law rather than the breadth of core public health activities. In contrast, local health departments appear to engage in abortion-related activities less often but, when they do, initiate a broader range of activities.

**Methods:**

This study aimed to: 1) describe the abortion-related activities undertaken by maternal and child health (MCH) and family planning professionals in state and local health departments; 2) understand how health departments approach their programmatic work on abortion, and 3) examine the facilitators and barriers to whether and how abortion work is implemented. Between November 2017 and June 2018, we conducted key informant interviews with 29 professionals working in 22 state and local health departments across the U.S. Interview data were thematically coded and analyzed using an iterative approach.

**Results:**

MCH and family planning professionals described a range of abortion-related activities undertaken within their health departments. We identified three approaches to this work: those mandated strictly by law or policy; those initiated when mandated by law but informed by public health principles (e.g., scientific accuracy, expert engagement, lack of bias, promoting access to care) in implementation; and those initiated by professionals within the department to meet identified needs. More state health departments engaged in activities when mandated, and more local health departments initiated activities based on identified needs. Key barriers and facilitators included political climate, funding opportunities and restrictions, and departmental leadership.

**Conclusions:**

Although state health departments are tasked with implementing legally-required abortion-related activities, some agencies bring public health principles to their mandated work. Efforts are needed to engage public health professionals in developing and implementing best practices around engaging in abortion-related activities.

## Background

State and local health departments are a critical part of the public health infrastructure in the United States, tasked with protecting and promoting the health of individuals and communities across a wide range of health issues. The specific roles and responsibilities of these agencies have evolved over time and, as a result, their organizational structure and authority varies considerably across level of government, geographic region, and area of public health [1]. Maternal and child health (MCH) and family planning are key programmatic areas of state and local health departments [2, 3] and have been studied extensively (e.g., [4, 5]). In contrast, while health departments have engaged with abortion for nearly 50 years [6], these activities have received much less scholarly attention.

### Health departments and abortion

The earliest governmental public health efforts related to abortion followed soon after its legalization and involved established public health tasks, including data surveillance and clinical quality improvement [6, 7]. In the late 1960s, the federal Centers for Disease Control and Prevention (CDC) established the national abortion surveillance system, based on reporting by and collaborations with state health departments. These data have been used to document the number and characteristics of women having legal abortions, as well as the safety of different procedures and care settings [8]. The CDC’s role in investigating abortion morbidity and mortality in collaboration with state and local health departments provided data for major judicial decisions and clinical improvements [9]. In 2016, 47 state health departments reported annual abortion data to the CDC’s surveillance system [10], and nearly all states report detailed abortion data on their departmental websites that are available to the public [11, 12].

The federal Title X Family Planning Program, first enacted in 1970, has also required health departments to engage with abortion. Title X grantees – which include some state and local health departments [13] – distribute funds to local clinics to deliver contraception, sexually transmitted infection, and other preventive services. Regulations have restricted Title X funds from paying for abortion services, but had required that pregnant women be offered non-directive information and counseling about their pregnancy options (including prenatal care, adoption and abortion) and be given referrals upon request [13, 14]. In 2019, the Trump Administration revised the regulations governing the Title X Program, prohibiting referrals to abortion and removing the requirement for pregnancy options counseling [15]. Title X grantees are responsible for ensuring these regulations are followed; thus, many state and local health departments have attended to abortion as part of their Title X activities for many years.

Over the past decade, health departments have taken on expanded roles in response to an increasing number of abortion-related policies enacted by state legislatures [16]. Some of these laws are antithetical to public health principles [17, 18]. For example, some state legislatures have tasked health departments with implementing regulations that single out abortion-providing facilities with requirements that are not mandated for facilities that offer other procedures of equivalent risk [19]; these regulations are not based in scientific evidence [20, 21]. These laws have resulted in facility closures that limit women’s ability to obtain abortion care [22, 23]. Some state legislatures have required health departments to produce and distribute health information materials for abortion-seeking patients that include scientifically inaccurate information, such as a disproven link between abortion and breast cancer [16].

This trend raises important questions about the use of the government public health infrastructure for the political purpose of impeding abortion care. Ideally, if health departments were to have a role in abortion, whether and how to engage in an abortion-related activity would be determined by identified needs and potential for positive impact on patients’ health [17]. Health departments would use established frameworks to guide and monitor the abortion-related activities provided in any public health jurisdiction, and would integrate abortion within the scope of their maternal and reproductive health activities. The CDC’s Essential Public Health Services (EPHS) framework, for example, describes and organizes the spectrum of public health tasks into ten types of core activities – such as monitoring the health status of the population, providing health information to the public, facilitating linkages to needed services, providing quality assurance, developing the public health workforce, and evaluating health services [24]. A public health approach to abortion would be informed by this type of broad-based framework and grounded in public health principles, such as basing policy and practice in the best available scientific evidence, assuring conditions in which people can be healthy, promoting health equity, meeting community needs, and assuring availability of health care (e.g., [1, 17, 18, 25–28].

### Examining abortion-related activities in health departments

Research on the abortion-related activities of health departments has been limited. Most is known about the history of federal and state involvement in abortion surveillance [6, 10]; much less is known about the programmatic abortion-related activities of state and local health departments. In a previous study, we systematically investigated the public-facing websites of state and local health departments to describe their activities related to abortion [11]. We coded all mentions of abortion on these website pages using the EPHS framework in order to understand the scope of these efforts. We found that most state health departments engage in some abortion-related activities; however, these largely reflect legal requirements rather than the range of core public health activities outlined by the EPHS framework. As expected, nearly all states conduct data surveillance and enforce some laws related to abortion. Activities to educate the public and provide referrals to services were mandated by legislation, rather than evidence-based health promotion goals. None of the state health departments were engaged in innovative research activities to develop best practices. We also found that few local health departments addressed abortion, although those that did engaged in a broader range of core public health activities.

The website study provided a useful window into understanding the typical abortion-related activities of state and local health departments, but did not provide an in-depth look at those activities, nor did it examine how the health departments approached mandated activities, or the reasons health departments took a particular approach. In the present study, we seek to better understand the abortion-related work of state and local health departments by interviewing public health professionals. Our specific aims were to: 1) describe the abortion-related activities undertaken by MCH and family planning professionals in state and local health departments; 2) understand how health departments approach their programmatic work on abortion, and 3) examine the facilitators and barriers to whether and how abortion work is implemented.

## Methods

### Participant recruitment

Between November 2017 and June 2018, we conducted key informant interviews with state and local health department employees based in MCH and/or family planning divisions. We chose these divisions because abortion would fit within their scope of service (in concept, if not in practice). We employed a purposeful sampling strategy to identify potential respondents. Respondents were eligible if they were currently working or had previously worked in MCH, family planning or an equivalent division within a state or local health department. We identified potential respondents through professional directories, professional conferences targeting state and local leaders, reviews of state and local health department websites, our team’s professional networks, and referrals from other respondents (i.e., snowball sampling).

We contacted potential respondents by phone and/or email to request their participation in the study. Prior to the interview, we sent all respondents an information sheet that described the study aims and procedures. We adjusted our recruitment strategies over the study period to capture a diverse geographic representation and balance between state and local health department representation in the final sample, so that we might examine findings by these key characteristics. We explicitly aimed to capture a range of experiences by geographic region and department level based on our understanding of differences in their roles and responsibilities [1].

We approached 66 individuals as potential respondents over the study period. Twenty-two agreed to participate and completed the interview, seven referred the interviewer to a different contact within their agency, 12 expressly declined participation, and 25 did not respond to the request. We tracked reasons for decline, which included lack of time and/or interest, lack of relevance to their work, unwillingness to take steps to receive approval from superiors, denial of agency permission, and concern over the political implications of the topic.

We considered recruitment complete when sufficient diversity in geographic and state/local representation had been achieved and no new themes emerged from interviews. A few respondents invited colleagues to participate in the interview to add other perspectives. As a result, the final sample for this analysis comprised 29 MCH/family planning respondents representing 22 health departments. All but one respondent was a current health department employee. The distribution of interviews by department type and geographic region is provided in Table 1.

**Table 1:** State and local health department respondents, by Census region

Health Department Type	Number of Respondents	Northeast	Midwest	South	West
State	12	3	2	3	4
Local	10	2	1	2	5
Total	22	5	3	5	9

### Study procedures

Interviews were semi-structured, following a general interview guide but allowing respondents to introduce topics that they thought were relevant to the discussion. The interview guide included questions about their department’s activities related to maternal and reproductive health care (including prenatal care, family planning, and abortion); the motivations for developing these activities; and the barriers and facilitators to integrating abortion into their department’s activities. Specifically, we asked about programmatic activities commonly undertaken by MCH and family planning divisions – interventions, programs, policies and tools – rather than data surveillance or facility regulation often done elsewhere in health departments. Questions were open-ended and modified over time to probe emerging themes. We note that these interviews were collected prior to the Trump Administration’s 2019 changes to the Title X Program [15]; therefore, responses may reflect prior Title X policies or activities that are no longer in effect.

One member of the study team conducted all interviews over the phone. Interviews lasted 30 to 90 min. Interviews were audio-recorded and transcribed verbatim, and field notes were written at the end of each interview. Respondents were offered a $50 gift card in appreciation for their participation. The study protocol was reviewed and deemed exempt by the institutional review board of the University of California, San Francisco.

### Analysis

The analysis used a hybrid approach to thematic analysis that included both deductive coding based on primary research aims and inductive coding of themes that emerged from the data [29, 30]. First, we categorized all abortion-related activities described by respondents using a previously developed codebook based on the CDC’s 10 Essential Public Health Services (EPHS) framework [11]. One author extracted all interview text describing abortion-related activities into a spreadsheet. The study team coded a short list of these activities using the extant codebook, discussed discrepancies, and revised the codebook. Three authors then independently coded all abortion-related activities, with at least two authors coding each activity. Together, the team resolved coding discrepancies and made final decisions about code application by consensus.

Next, two authors independently reviewed a subset of interview transcripts and developed preliminary thematic codes regarding the approaches to, barriers to, and facilitators of abortion-related activities. These were revised through discussion and applied to all transcripts using Dedoose qualitative data management software (SocioCultural Research Consultants, 2016). The first author analyzed the coded data for thematic patterns, including commonalities and differences across interviews. We examined how themes varied across respondent characteristics, specifically department level and geographic region. The quotations presented indicate whether the respondent was from a state or local health department and their region, except in cases where we were concerned a health department could be identified. All members of the team reviewed all transcripts and provided ongoing input on the analysis. COREQ guidelines for the reporting of qualitative research were used to guide the presentation of these methods and results [31].

## Results

### Abortion-related activities within health departments

Nearly all respondents – at 11 of 12 state health departments and all 10 local health departments – described some abortion-related activities taking place within their agencies. Examples of the range of activities in health departments: collecting data from abortion providers and preparing analytic reports; developing state-mandated materials for abortion-seeking patients; developing policies related to abortion care, referrals and funding; enforcing federal and state laws regarding payment for abortion; facilitating linkages to abortion and/or non-abortion services (e.g., pregnancy resource centers, prenatal care, adoption assistance); training abortion providers on administrative policies; and monitoring Title X family planning providers’ provision of pregnancy options counseling. Table 2 presents a summary of the types of activities described, categorized according to the EPHS framework.

**Table 2:** Abortion-related activities described by state and local health department respondents, categorized by Essential Public Health Services (EPHS) framework (*N* = 22)

	# State (***n*** = 12)	# Local (***n*** = 10)
**EPHS1 - Monitor health status to identify and solve community health problems**	3	1
• Collect abortion data from facilities (“induced termination of pregnancy” forms) • Prepare regular surveillance reports (e.g., for state legislature)		
**EPHS2 - Diagnose and investigate health problems and health hazards in the community**	0	0
*(No examples provided)*		
**EPHS3 - Inform, educate and empower people about health issues**	5	0
• Develop and update state-mandated abortion information, consent forms, and websites (“Women’s Right to Know”)		
**EPHS4 - Mobilize community partnerships to identify and solve health problems**	2	2
• Convene provider workgroups to address availability and provision • Partner with community and social service agencies to address availability and referrals • Develop inter-agency partnerships to address referrals		
**EPHS5 - Develop policies and plans that support individual and community health efforts**	3	5
• Develop internal policies related to pregnancy options counseling and abortion referrals • Develop administrative policies and systems for Medicaid or other state coverage of abortion (e.g., enrollment forms, billing processes) • Review policies to understand extent that abortion-related services are allowed by law		
**EPHS6 - Enforce laws and regulations that protect health and ensure safety**	11	6
• Enforce federal/state requirements regarding funding for abortion • Implement state laws that allow use of public funding to pay for abortions • Develop and update state-mandated abortion information, consent forms, and websites • Collect mandated data and prepare reports on abortion-related topics, as required by law • Conduct research and provide legislative testimony on abortion-related policies as requested by legislature • Maintain resource directories for abortion services and/or alternatives to abortion, as required by law • Implement laws that require the provision of abortion alternatives (e.g., funding for CPCs)		
**EPHS7 - Link people to personal health services and assure the provision of health care when otherwise unavailable**	7	10
• Provide pregnancy options counseling (e.g., at Title X clinics) • Facilitate linkages for women seeking abortion services (e.g. information guides, case management, insurance coverage, funding, transportation, childcare) • Provide resources linking to alternatives to abortion (e,g., CPCs, hotlines) • Provide direct abortion services at clinic/hospital • Pay for abortion services through state public funding		
**EPHS8 - Assure a competent public and personal healthcare workforce**	1	4
• Train Title X providers about pregnancy options counseling and referrals • Train abortion providers on Medicaid policies, billing, reimbursement, presumptive eligibility, etc. • Train local clinic staff and programs about abortion (generally), pregnancy options counseling, and referrals		
**EPHS9 - Evaluate effectiveness, accessibility and quality of personal and population-based health services**	6	2
• Conduct quality assurance monitoring of pregnancy options counseling at Title X clinics • Conduct quality assurance monitoring of publicly-funded CPCs • Monitor separation of service provision (abortion vs. other family planning) • Develop standards of care/certification requirements for abortion services • Conduct assessment of local abortion access • Collect and analyze abortion data		
**EPHS10 - Research for new insights and innovative solutions to health problems**	0	0
*(No examples provided)*		

Respondents in state health departments described abortion-related activities across 8 of 10 categories in the EPHS framework. The most common Essential Services for state health departments were those that Enforce Laws (EPHS6, 11 respondents), Link to Services (EPHS7, 7 respondents), and Evaluate Effectiveness, Accessibility and Quality (EPHS9, 6 respondents). Respondents in local health departments described activities across 7 of 10 Essential Services. The most common were activities that Link to Services (EPHS7, 10 respondents), Enforce Laws (EPHS6, 6 respondents), and Develop Policies (EPHS5, 5 respondents). None of the state or local respondents described activities relating to EPHS2 (Diagnose or Investigate) or EPHS10 (Innovative Research); in addition, none of the local respondents described EPHS3 activities (Inform and Educate).

One state health department reported no abortion-related activities. Others reported very few activities. To some extent, this may reflect that MCH and family planning divisions are not necessarily involved in the entire range of abortion-related work in which a health department engages. As one state respondent in the Midwest explained, “*We’re a really big health department, so there could be [other] people. There probably is a whole licensing division that licenses the facilities, but I don’t work them closely.”* A state respondent in the West made a similar point about the department’s Medicaid division, which was responsible for the implementation of legislation and activities around use of state Medicaid funds to pay for abortion.

Some respondents asserted that the lack of abortion-related work in their department was not deliberate; as one state respondent in the Midwest noted, abortion had simply never come up in discussions: “*It’s not that there is an intentional effort to not talk about or deal with or think about it.”* A respondent from another state health department in the Northeast that engaged in few abortion-related activities similarly noted, “*It has never come up. I’m trying to even imagine in what universe that would come up*.”

### How health departments approach abortion-related activities

Respondents described the impetus for the abortion-related activities undertaken within their divisions of their health department. Based on our prior research [11], which indicated that legislative mandate was a strong driver of abortion-related work, we thematically coded each department’s activities based on the extent to which efforts were driven by legislative mandate or initiated by the department. During this analysis, we identified a third scenario – a middle ground – in which MCH and family planning professionals in health departments found flexibility as they implemented mandated activities. In this section, we describe a continuum of approaches to abortion-related work. We provide examples of how health departments went about their abortion-related work in three categories: 1) executing mandated activities as prescribed, 2) bringing a public health approach to mandated activities, and 3) initiating public health focused efforts without mandate to meet identified needs.

The distribution of these categories is presented in Table 3. Some respondents described more than one approach to abortion-related work; therefore, a health department may fall into more than one category. This indicates that, within some departments, different activities were undertaken for different reasons or approached in different ways.

**Table 3:** Types of abortion-related activities in state and local health departments, by category

Health Department Type	Number of Respondents	Category 1: Executing mandated abortion activities as prescribed	Category 2: Bringing a public health approach to mandated activities	Category 3: Initiating public health approaches without mandate	No Abortion-Related Activities
State	12	11	5	2	1
Local	10	4	0	8	0

#### Category 1: Executing mandated activities as prescribed

Respondents from 14 of 22 health departments described engaging in abortion-related activities that were executed only when required by law – and only in the ways prescribed by the law. This was particularly common for state health departments (11 of 12 state respondents) but also reported by a few local health departments (3 of 10). Respondents often cited the specific laws and regulations that dictated their abortion-related work.

For example, as respondents in MCH and family planning divisions, many respondents described their responsibility for ensuring that clinic sites met the legal requirements of the federal Title X Family Planning Program. One state respondent in the West described being “*vigilant*” about ensuring that pregnancy options counseling be made available at their Title X family planning clinic sites, as required (at the time of the interview):

*“The first thing that we do according to Title X – and I can cut and paste that regulation for you – is that we offer options counseling for women who request it. That, of course, is something all of our sites do. And we check up every site that we have … We follow up to make sure that happens.”*

Another state respondent in the South explained their department’s legal responsibility in ensuring compliance with policies regarding how (and how not) to make referrals to abortion providers:

*“To the extent that we discuss [abortion] is within the range of a general understanding that that is an option …*. *All we can do, legally, as a Title X site – by federal mandate – is say ‘Well, if you want an abortion, here’s a number to contact. You have to set up the appointment.’ We literally cannot do it. We are, by funding, not allowed to do it [for her].*

Some state respondents described activities to develop information for people seeking abortion, either for public availability on the health department website or direct distribution to patients at the abortion clinic appointment. For some of these states, the process of developing and distributing information materials was strictly prescribed. State legislation dictated the specific information that the health department had to include in the materials: *“It’s all spelled out in the law... You have to read about your procedure, you have to look at an image of a fetus at whatever week you might be in, and you [have to] print out a form at the end and sign it.”* Other examples of mandated activities included reporting of abortion data to the state legislature, developing certification requirements for abortion providers being reimbursed by the state, and executing policies regarding state insurance coverage for abortion care.

#### Category 2: Bringing a public health approach to mandated activities

Five of 22 respondents – all in state health departments – described engaging in abortion-related activities that were initiated when mandated by law, but implemented with some amount of flexibility. In these cases, health department respondents described taking an active role in decision-making around how to implement the required abortion-related activities. They met legal or funding requirements and also incorporated common public health principles – an emphasis on scientific accuracy, clinical expert engagement, presenting unbiased and neutral information, and/or promoting access to care – into the department’s abortion-related work.

The most common activity under this theme was the development of state-mandated information materials about abortion. Some states described examples where all or some of the content of websites and/or patient materials regarding abortion was left to the discretion of the health department. In such cases, health departments often sought outside clinical expertise (e.g., from medical boards, nurse consultants, obstetrician/gynecologists), working to ensure that materials reflected the best available scientific evidence and clinical expertise. A state respondent in the Northeast said:

*“The legislature passed a law that required a state-mandated consent form for abortion, and tasked … the department of public health with developing this form …*. *And so [the department] brought together a group of abortion providers … to help write this consent form, to make sure it was accurate and appropriate, and useful to providers.”*

Another state respondent in the West similarly described working with the state medical board to ensure that the information on its mandated website was both “*scientifically based and … unbiased in its approach.”* Presenting unbiased and neutral information was an explicit goal, as members of the medical board and the health department held politically diverse opinions about abortion. Together, they *“agreed on the common ground that it’s not about what they believed, that it was about what was the best information to be providing to a woman making a choice about her pregnancy and helping her for truly informed consent.”* The same department developed an online resource guide for pregnant women – mandated generally by state law, but approached using public health principles to include a broader range of services than required by the state. The resulting guide included information about where to seek prenatal care, pregnancy support, faith-based social services, health services, as well as family planning and abortion care.

In general, respondents felt positively about bringing public health principles to the implementation of the state requirements, seeing it as “*fortunate*” that the health department could “*take the legislation that could have gone otherwise*” if not guided by the health department. One state respondent in the Midwest noted that their division within the health department “*volunteered, actually, because we wanted to … make sure it was done appropriately and with accuracy.”* Another in the West agreed:

“*I was just so pleased that we took the legislation that could have really been harmful to women’s access... I was really glad to see that we were allowed to make it something that was actually useful and met everybody’s needs.”*

In a few cases, the efforts to bring public health principles to legal or funding requirements resulted in ongoing health department engagement around abortion. For example, for one state department in the Northeast, a state-mandated information requirement was the impetus for convening a working group of abortion providers, but it created an opportunity for the health department to think more comprehensively about their abortion work. The department continued to convene the provider group to help identify clinic training needs, barriers to access, and programmatic and policy priorities related to abortion.

#### Category 3: Initiating public health approaches to abortion without mandate

Ten of 22 respondents described initiating abortion-related activities that were not prompted by a legal or funding requirement but by identified need. Department-initiated efforts were much more common for local health departments (8 of 10) than state health departments (2 of 12). Often, departments aimed to incorporate abortion into ongoing activities around pregnancy-related care.

Respondents described varied activities related to abortion, although many examples focused on improving referrals to abortion services. One local health department began offering pregnancy options counseling and referrals to high school students using the department’s mobile clinic. Respondents in two separate local health departments described wanting to ensure “*a warm handoff*” for patients receiving a positive pregnancy test at their public family planning clinics. As one local respondent in the South explained, *“It is one thing to hand a patient a few papers and say ‘go do this.’ It’s another thing to [say], ‘Let me really link you with this person.”*

A few health departments initiated activities to reach marginalized populations with information on how to access abortion care. One state department in the West began by asking *“Where are we going to find folks who might benefit from the services?”* and built networks with social service providers, community action organizations, and groups working with migrant farmers to reach undocumented immigrants who might be seeking abortion. A local respondent in the West described working with their department’s home visiting program to ensure that women enrolled in the program received unbiased counseling and referrals about their pregnancy options:

“*[We want to make sure staff are] not just finding that she’s pregnant and saying, “Great! You’re pregnant! How can I help you to have a healthy baby?’ but ‘So, you’re pregnant. What does that mean for you? What do you need? Let’s have a conversation about it. How’s your mental health? What kind of referrals can I give you?”*

Other department-initiated abortion activities focused on expanding clinical services, including: providing abortion services in department outpatient clinics and hospitals, improving the quality of post-abortion contraceptive care, working with community health centers to expand access to medication abortion, and planning for potential increases in abortion patient volume if neighboring states enact restrictive abortion policies. One state health department in the West began collecting and reporting on abortion data, not because it was required by law, but because they did so for other, similar public health issues of interest:

“*It’s not mandated, however, that we take the data and report on it. That is something that’s kind of born from our shop. I think that’s important. That a public health department is actually interested in the abortion data – tracking it and reporting, kind of making it part of the story, making it part of the narrative [of family planning success].”*

Initiating new abortion-related activities often required convincing other health department colleagues of abortion’s relevance. As one local respondent in the Northeast described:

*“We had special symbols on how to find places in the [resource] guide. And one of them was a symbol for abortion care. I remember being in a meeting with someone who worked in another part of [the city], and they were just surprised that there was abortion in there. And they were like, ‘Do you really need that?’ And I was like, ‘Yeah, you really need that.’”*

### Facilitators of and barriers to abortion-related activities

Across all three categories of approaches, respondents described factors that affected both whether and how their departments engaged in abortion-related activities. These facilitators and barriers were related; that is, the overarching factors that support abortion work in one health department hamper it another. We describe these briefly.

#### Political climate

Many health department respondents discussed the state or local political climate as either a facilitating or restrictive factor in engaging in abortion-related activities. Even in the absence of specific laws mandating health departments to engage with abortion in particular ways, they felt the impact of the environment in which they operate. For one state respondent, conservative state politics keeps the department from initiating abortion-related activities, especially those that facilitate access:

“*I think the political climate here would probably not promote any abortion-related services …*. *Even in the absence of Title X regulation putting a prohibition on it, I don’t think that the state would touch it with a ten-foot pole.”*

In contrast, other respondents operated in a political environment where leaders supported abortion rights and gave the department freedom to implement abortion-related activities that align with public health principles. One local respondent in the West described their department as “*very lucky”* for being able to participate in campaigns that promote access to abortion, supported both by the health department director and the city’s mayor: “*I think we have the luxury to do that here in a way that you wouldn’t [elsewhere].”*A few respondents described the need to take in account the range of political opinions across their state when initiating or considering how to implement abortion-related work. A state respondent in the West explained:

“*We’re diverse, politically. We are very progressive, [but] we have an incredibly conservative side of the state … It’s something we’re challenged with and just have to work with. We can’t just assume that everybody in our state thinks the way we do.”*

#### Funding

Many respondents noted that whether and how their department engaged with abortion was affected by the specific requirements of federal and state funding sources. One state health department respondent in the Midwest noted:

*“Our [department’s] major program is the Title X Family Planning Program … .and that has such a separation from abortion that I think that everybody’s just really careful to try to not get too involved in abortion services. Because we want to be compliant with our funding source through that program.”*

For some respondents, at both the state and local level, fear of losing existing funding due to restrictions on Title X funding led to trepidation about engaging in abortion-related activities, as they feared it could jeopardize funding for their entire program. One local respondent in the South described: *“anything [that may] screw with our Title X funding is a terrible idea.”* This was most commonly expressed in relation to Title X funding, but was described about other sources as well, such as the Title V Maternal and Child Health Block Grant.

Respondents also discussed feeling constrained by specific demands of funding, which gave limited room for creativity, flexibility, or innovation in whether they initiated their own activities or how they implemented mandated activities. Since no federal funding and little state funding is specified for or inclusive of facilitating access to abortion, the department has no mechanism through which to explore including abortion in their work. A state respondent in the South noted:

*“A lot of times, you just are kind of siloed to what the funders are asking you, rather than having just a general discussion of what are the needs of the community, and how can we manage our programs to better fit those needs of the community.”*

In contrast, respondents based in departments that received funding from diverse funding streams and those that did not receive Title X funds described feeling less constrained by funding. A few described the opportunities that came with being in a state that provides insurance coverage for abortion care with public funds, and a few had access to funding specifically for initiating abortion-related activities. One local respondent in the West described being able to initiate a public information campaign about availability of abortion services with the receipt of dedicated internal funding:

*“I think [abortion] was always kind of under the surface, but not a priority. And then it became a priority. And then we were able to do things about it because there was a windfall of money, or we had some savings …*. *I thought ‘Let’s spend it on this campaign.’ And I had support from the staff to do that. I was not taking money away from any other program. That might have been really a hot button.”*

#### Departmental leadership

Respondents believed that individual leadership within their department could nudge a department toward or away from bringing a public health approach to abortion-related activities. One local respondent in the West described an environment where division staff were open to the idea of addressing abortion in their work, but did not have the support of leadership to develop or implement ideas. When the leadership changed, abortion *“became something that we did, and also talk [ed] about more.”*

Having department or division leadership that prioritized “*an evidence-based approach to public health,*” innovation and, more specifically, inclusion of abortion in public health, was also described as crucial to facilitating abortion-related activities. This was particularly true when the political climate might be less supportive. A state respondent noted:

*“I am eternally grateful to be doing this work [on abortion here]. I can count on the support of my boss and my boss’s boss, and her boss, who’s the Commissioner …*. *And it’s not that we haven’t had troubles with this; we have anti-choice state legislators that are trying to pass anti-choice laws every year.”*

In contrast, departmental leadership that was opposed to abortion or did not want to initiate new public health oriented activities related to abortion was seen as a formidable barrier, even in states with supportive policy environments for abortion. Proposing new activities related to abortion, one state respondent noted, *“wouldn’t make it past our division chief.”* A local respondent in the Northeast described the challenge of changing the minds of long-term staff about the department’s involvement in sensitive subjects like abortion: “*It’s like putting a crack in a boulder. Somebody has to keep hitting it over and over and over again …*. *[Our greatest success has been] where there are one or two people who are just amazing and put in heroic amounts of effort and time into it.”*

## Discussion

In accounts of the roles of public health professionals in health departments, there are typically two poles of work described: one that requires implementing laws and upholding the public health bureaucracy, and the other that involves initiating activities to improve and advocate for changes in social, policy, and environmental factors that adversely impact health [32]. Consistent with this framework and our previous research [11], this study found evidence of the strong influence of federal and state policies on the abortion-related activities undertaken within MCH or family planning divisions of health departments. In a time of increasing state legislation around abortion [16], it is not surprising to find that that state health departments, in particular, are implementing abortion-related activities dictated by law. Fundamentally, implementing and enforcing laws and policies is a key responsibility of health departments. We also found clear evidence that some health departments, particularly local health departments, initiate abortion-related activities – such as facilitating linkages to abortion services and assuring the provision of abortion in the community – guided by core public health principles and frameworks (e.g., [17, 24, 26–28].

A key finding of our in-depth interviews – one not identified on public-facing websites and not addressed in frameworks that describe polarities of public health practice – is that, even in the context of legally required activities, some health departments found room to incorporate public health principles. For example, study respondents described bringing research evidence to mandated activities and convening clinical experts to ensure products produced would both be evidence based and meet their patient care needs. One concrete example was the development of state-mandated information (“Women’s Right to Know”) materials distributed to women presenting for abortion. In some states, the details for these materials were formally stipulated, and the health department’s role was solely to implement the law as written, even if this necessitated including inaccurate information. In other states, however, the law afforded enough flexibility for health department professionals to bring their public health expertise and training to their task. In these cases, respondents spoke of ensuring materials were evidence-based and language was unbiased, engaging clinical experts in the development process, and aiming to make materials useful for providers and patients alike. From this, we conclude that the implementation of mandated abortion-related activities can – at times – be guided by the frameworks, principles and values core to the public health profession. Our findings suggest that regardless of political climate, public health professionals in health departments have a range of options to bring public health principles to abortion-related activities. Further exploration is needed to understand the factors that allow them to do so, especially in circumstances where the abortion-related activities are mandated by the state legislature.

This study has limitations. First, our flexible interview guide allowed for deep exploration, but limited our ability to make comparisons across the entire sample. Second, our findings are limited by the knowledge and experiences of our specific respondents, as well their willingness to share them with us. As noted by respondents, activities taking place elsewhere in the health department may not be known to those in the MCH or family planning divisions. This likely explains the undercount of vital statistics collection presented in Table 2 (EPHS 1) compared to prior research [8, 10, 20]. Third, due to the scope of the study, we did not explore differences by respondents’ placement within a MCH vs. family planning division, which could affect their engagement with abortion. Fourth, abortion is a politically sensitive topic. It is possible that our respondents were not forthcoming when asked about the facilitators of and barriers to engaging with abortion in their department, despite assurances of confidentiality. Finally, while we reached out to health department officials across states and localities, many potential respondents declined to participate. This may be due to actual or perceived political constraints, departmental policies about engaging in research, or personal comfort talking about abortion. While we did have respondents from a range of geographies and political climates among our sample, the findings may have limited applicability to other health agencies or jurisdictions. In particular, further research is needed to understand the influence of the state abortion policy environment on local health departments, especially in situations where state and local politics do not align.

This study also has considerable strengths. To our knowledge, this is the first study that includes perspectives of public health professionals to understand whether and how health departments engage with abortion. Research on the role of health departments in the availability and provision of abortion is minimal; our studies begin to fill that gap. This paper, in particular, adds the voices and experiences of MCH and family planning professionals themselves – a rich source that provides insight beyond that of publicly available websites, materials, or statutes.

## Conclusions

This study finds that MCH and family planning professionals in health departments are engaging in a range of abortion-related activities. Much of this work is responsive to federal and state requirements, rather than initiated and guided by core public health frameworks, principles, and values. Nonetheless, there is compelling evidence that some health departments – at both the state and local level and in diverse political settings – are able to bring a public health approach to abortion-related activities. New efforts are needed to engage public health professionals in developing and implementing best practices around abortion-related activities.

## Data Availability

The datasets generated and/or analyzed during the current study are not publicly available due to the terms of participant consent but are available from the corresponding author on reasonable request.

## Abbreviations

CDC: Centers for Disease Control and Prevention
EPHS: Essential Public Health Services framework
MCH: Maternal and child health
US: United States
